# Impact of an educational intervention on pharmacovigilance knowledge and attitudes among health professionals in a Nepal cancer hospital

**DOI:** 10.1186/s12909-020-02084-7

**Published:** 2020-06-03

**Authors:** Sunil Shrestha, Sabina Sharma, Ramesh Bhasima, Puskar Kunwor, Baburam Adhikari, Binaya Sapkota

**Affiliations:** 1 Pharmacy, Nepal Cancer Hospital and Research Center, Harisidhhi, Lalitpur, Nepal; 2 Clinical Pharmacy Unit, Nepal Cancer Hospital and Research Center, Harisidhhi, Lalitpur, Nepal; 3 Department of Pharmaceutical Sciences, Nobel College, Affiliated to Pokhara University, Sinamangal, Kathmandu, Nepal

**Keywords:** Adverse drug reactions, Drug safety, Educational intervention, Healthcare professionals, Nurses, Nepal, Pharmacists, Pharmacovigilance

## Abstract

**Background:**

Pharmacovigilance (PV) knowledge and attitudes among health care professionals (HCPs) directly affect the practice of PV and reporting of adverse drug reactions (ADRs) in a hospital. The main aim of the study was to assess the impact of an education intervention on the knowledge and attitude of HCPs attached to the regional PV center in an oncology based hospital of Nepal.

**Methods:**

A pre-post intervention questionnaire-based study was conducted amongst HCPs, i.e. nurses and pharmacists of an oncology based hospital of Nepal. A 21-item pretested PV questionnaire (twelve and four statements for assessing knowledge and attitude respectively) was used before and after an educational intervention among 89 HCPs working in the hospital. SPSS version 21.0 was used to analyze pre- and post-survey data with descriptive and inferential statistics. The knowledge and attitude scores before and after educational intervention were analyzed with Wilcoxon rank-sum test.

**Results:**

After the educational intervention, the mean score for knowledge about PV and ADR increased from [mean ± S.D] 6.90 ± 2.527 (pre-test) to 11.36 ± 1.189 (post-test, *p* < 0.001). There were significant improvements after an educational intervention in attitude from 1.80 ± 0.932 (pre-test) to 3.61 ± 0.556 (post-test, *p* < 0.001). Twenty-one (23.6%) of participants responded that they lacked the time to report ADR, while 12 (13.5%) replied that there was no remuneration for reporting ADR. Approximately 24% of them recommended frequent pharmacovigilance awareness programs via continuing medical education or other similar kinds of workshops for improvement of pharmacovigilance.

**Conclusion:**

The preliminary findings of this study showed that knowledge and attitude scores were increased following an educational intervention. They also suggested that training on PV and ADR tends to have a positive impact on knowledge and attitude among nurses and pharmacists working at an oncology based hospital in Nepal.

## Background

Patient’s wellbeing and safety while taking medications are an important issue for all healthcare professionals (HCPs). An adverse drug reaction (ADR) is defined as “a *response to a drug which is noxious and unintended, and which occurs at doses normally used in man for the prophylaxis, diagnosis, or therapy of disease, or for the modifications of physiological function”* [1]. Pharmacovigilance (PV) is an organized procedure for observing and identifying ADR [2], and is defined as “*the science and activities relating to the detection, assessment, understanding and prevention of adverse effects or any other drug-related problem*” [3].

For example, PV of antineoplastic agents is necessary for the safety of lives and to utilize available health resources cost-effectively [4]. In clinical practice, both from clinical and economic points of view, ADRs may have a dramatic influence [5, 6]. ADR sometimes causes an additional treatment while managing ADRs leading to the economic burden, which can be minimized by ADR reporting [5, 7]. Underreporting of ADRs confines and interrupts initiatives that could have been taken to avoid or lessen the harmful effects of medicines [7]. Furthermore, not notifying and reporting harmful effects of a drug even after experiencing it, is not ethical. This might intentionally place the risk to other patients or consumers with the same medicines [8]. Quick reporting of ADRs is reasonable and debatably, the greatest technique for drug safety surveillance [9]. ADR reporting and PV have significant public health implications as these contribute to the prevention of future similar types of ADRs, possibly saving lives and reducing the economic burden.

In Nepal, the Department of Drug Administration (DDA) is a National Medicine Regulatory Authority. DDA was appointed as a national PV center by the Nepal Government to liaise with the World Health Organization (WHO) Program for International Drug Monitoring (IDM) in the year 2004 [10]. The primary function of the DDA is to gather case reports from 15 regional PV centers in Nepal. The Nepal Cancer Hospital and Research Center (NCHRC) is one of these regional PV centers and was the first cancer hospital in Nepal to hold that distinction [11]. Globally, the effectiveness of a PV program can be determined by the active involvement of HCPs, and their knowledge, attitude and practice (KAP). Education and training programs can improve the KAP of HCPs regarding PV and ADR reporting [12–15]. Before conducting any kind of intervention such as educational training, it is important to assess the reference point of KAP of the HCPs concerning ADR monitoring and PV, to better design the training and determine its impact. The awareness, knowledge, and attitudes of the HCPs regarding PV and its existence in the country directly affect the practice of PV and ADR reporting in the hospital. In the context of Nepal, underreporting of ADR is a problem [16]; the awareness of HCPs about ADR reporting and PV in the country may ultimately improve ADR reporting [17] and it should be strengthened in every hospital [18]. In the 16 years of DDA’s work, the National database contains 547 ADR reports [10]. Several studies in Nepal have reported that HCPs have a lack of knowledge regarding the PV before any kind of educational intervention [14, 15, 17, 19, 20]. Few studies, however, conducted in Nepal to assess the outcomes of an educational intervention to increase KAP of HCPs [21–23]. Educational intervention, interactive sessions and multifaceted continuous medical education support knowledge and behavioral changes in health professionals [24, 25]. Pre-post studies conducted at a different institution had also suggested the positive impact of education on ADR and PV activities [26, 27]. The educational intervention is rooted in an approach to social learning theory [28], which includes the construct of self-efficacy of HCPs as an important component of professional behavior change. In short, self-efficacy comes from HCPs’ perceptions that they could make changes in their practice, specifically to report ADR.

With this background, the main objective of the present study was to assess the impact of an educational intervention on the knowledge of PV and ADR reporting, attitude towards reporting ADR among HCPs (nurses and pharmacists) working in the NCHRC.

## Methods

### Study design

The study was a pre-post intervention study.

### Study site

NCHRC is a 100-bed oncology hospital in Harisiddhi, Lalitpur, Nepal, that provides comprehensive cancer care such as chemotherapy, radiotherapy, and surgery. NCHRC is the first hospital cancer hospital in Nepal to serve as a regional PV center [11].

### Sampling technique and sample size

Slovin’s formula [29] was used to calculate the sample size, based on the total number of nurses and pharmacists working at the study site during a period of the study and 5% of margin error was assumed. The least sample size needed to be for the study was calculated as 79 and the total sample size was increased to 89 in consideration of 10% of the non-response rate. It was determined to study a convenience sample of nurses and pharmacists for this study, and sample doctors, medical oncologists were excluded from this study for subsequent studies.

### Study instrument

Based on prior related studies reported in various literature [12, 15, 19, 21–23, 26, 30], a questionnaire was developed and subjected to a review and validation process. The developed questionnaire was then verified for readability and ease of accepting among 10 HCPs who were working in a different hospital with similar settings to the study site. Face validation of the data collection instrument was carried out by coworkers from the nursing and pharmacy departments. Content validation of the instrument was completed by referring the questionnaire to content experts of PV, hospital pharmacy, clinical pharmacy pharmacology, and statistician within the country. Cronbach’s alpha value was calculated to measure internal consistency which was found to be 0.76, signifying good internal consistency. The findings of the pilot study were not included in the final data analysis except for the modification of the questionnaire.

The final version of the pre-study instrument included four sections. Section 1 comprised of 5 items questionnaire that explored demographic and related information of HCPs: age, gender, working experience, educational qualifications and professional status. Section 2 comprised of 12 items designed to evaluate HCPs’ knowledge regarding PV and ADR. Section 3 comprised of 4 items to evaluate the attitude and section 4 comprised of 10 items to evaluate HCP’s current practice related to PV.

The final version of the post-study instrument also included five sections. Section 1 comprised of 5 items questionnaire that explored demographic and related information of HCPs: age, gender, working experience, educational qualifications and professional status. Section 2 comprised of 12 items designed to evaluate HCPs’ knowledge regarding PV and ADR. Section 3 comprised of 4 items designed to evaluate attitude. Section 4 and 5 comprised of two open-ended questions to the participants to assess the discouraging factors for underreporting of ADRs and to get feedback from the participants as a recommendation for improvement of ADR reporting in future. Practice related questions were not included in the post-study instrument as we wanted to assess the PV and ADR reporting practice before any kind of educational intervention.

### Process of data collection

HCPs were approached and the aim of the study was explained. Written informed consent from the participants was taken and was assured that their participation in this study was voluntary and confidentiality will be maintained. The questionnaire was distributed to 89 HCPs, before the PV training, and they were given 30 min to complete and return them to one of the pharmacist investigators for the study. Any explanation when required, while filling up the questionnaire and further time to complete the given questionnaire was given to the participants when required. During the pre-test, those nurses and pharmacists who were busy during that time were requested to complete and return the questionnaire in their free time, before the educational training.

### Educational intervention (Training in PV and ADRs)

After completion and collection of pre-survey responses from all 89 participants, training in PV and ADRs was conducted. First of all, an introduction was given to the participants regarding the objectives of training on “pharmacovigilance and ADR”. The training was given by the PV team including coordinator of PV for the hospital, a senior clinical pharmacist and a senior hospital pharmacist. The teaching-learning aids used during training were PowerPoint presentations, lectures, posters. Handouts were also given and ADR reporting form which was developed by regional PV was also demonstrated to the participants. The training was followed by interactive discussion, questions and answers. The training comprised an overview of ADRs and PV including the definition of PV and ADR, types of ADRs, history of PV and PV network of Nepal, introduction to National and Regional PV centers of Nepal. ADR reporting form developed by NCHRC was introduced to the participants and how to fill ADR reporting form was also discussed. In the training, the role of HCPs in PV was also discussed. Similarly, the demonstration of Vigiflow was given to all the participants, which is a web-based individual case safety report (ICSR) management system, used for recording, processing and sharing reports of adverse effects. It is accessible for practice by national PV centers of the WHO program for IDM [31].

The duration of the interactive training was around 2 h. After training, all participants were administered the post-test questionnaire.

### Data analysis

Pre-test and post-test responses were thoroughly checked for errors. The mean score was calculated for assessing the pre-survey KAP level among participants. The responses to attitude questions of the Likert scale were joined into combinations of (Agreement) “strongly agree” and “agree” and (Disagreement) “neutral”, “disagree”, and “strongly disagree”. Agreement and disagreement were scored 1 and 0 respectively. Knowledge and attitude mean score of HCPs before and after an educational intervention was also compared and analyzed with the Wilcoxon rank-sum test, using IBM SPSS for Windows, Version 21.0. Armonk, NY: IBM Corp. The *p*-value of less than 0.05 was considered statistically significant.

## Results

### Socio-demographics of healthcare professionals

Eight-nine of invited HCPs (nurses and pharmacists) participated. Table 1 shows the demographic characteristics of the participants. Seventy-Eight (87.6%) was in the age group of 21–30 years. The vast majority of participants were female 76 (85.4%) and 13 (14.6%) were male. Among participants, 55(61.8%) were staff nurses, 12(13.5%) were nurses, 15(16.9%) were assistant pharmacists, 5(5.8%) were pharmacists working at outpatient and inpatient pharmacies of the hospital and only 2(2.2%) were clinical pharmacists. Staff nurses hold a qualification diploma and nurses hold a qualification of bachelor’s in nursing. The majority of participants’ educational qualifications were of 48(53.9%) diploma degree (staff nursing or a diploma in pharmacy), 36(40.4%) bachelor degree (Bachelor in nursing or Bachelor in pharmacy) and 5 were graduate degrees (Masters in Nursing or Masters in Clinical Pharmacy or Doctor of Pharmacy). Almost 90 % of the participant had work experience of 0.2–5.1 years and 1(1.1%) participant had work experience of more than 15 years (Shown in Table 1).

**Table 1:** Demographic characteristics of the participants

Study variables	Frequency (n)	Percentage (%)
Age (in years): (25.88 ± 4.131) (Range: 20–46)		
< = 20	4	4.5
21–30	78	87.6
31–40	5	5.6
41+	2	2.2
Total	89	100.0
Gender		
Male	13	14.6
Female	76	85.4
Total	89	100.0
Professional Status		
Staff nurse	55	61.8
Nurse	12	13.5
Assistant pharmacist	15	16.9
Pharmacist	5	5.6
Clinical Pharmacist	2	2.2
Total	89	100.0
Department		
Nursing	66	74.2
Pharmacy and Clinical Pharmacy	23	25.8
Total	89	100.0
Qualifications of the participants		
Diploma Degree	48	53.9
Undergraduate	36	40.4
Graduate	5	5.6
Total	89	100.0
**Working experience (in years):** (Mean ± SD: 2.837 ± 3.0177) (Range: 0.1–18)		
< = 0.1	1	1.1
0.2–5.1	80	89.9
5.2–10.1	5	5.6
10.2–15.1	2	2.2
15.2+	1	1.1
Total	89	100.0

### Practice of the pharmacovigilance without any educational intervention

Table 2 shows the practice related statements of the ongoing pharmacovigilance activities and ADR reporting among HCPs before any kind of educational intervention or training in the hospital.

**Table 2:** Practice related statements regarding pharmacovigilance without any educational intervention

Practice questions	Yes (n, %)
Experienced ADR in a patient during the profession	47 (52.8)
Seen ADR reporting form	30 (33.7)
Reported ADR to PV center	11 (12.45)
Trained on how to report ADR	20 (22.5)
PV committee in the Institute	35 (39.3)
Keeping records of ADR	56 (62.9)
Visited any ADR Monitoring Center	19 (21.3)
Will report ADR if occurs in future	70 (78.7)
Read an article on prevention of ADR	38 (42.7)

### Comparison of pre response and post response regarding knowledge and attitude related questions

Table 3 shows a comparison of responses before and after the training on pharmacovigilance and ADRs regarding knowledge and attitude related questions. A significant difference (*p* < 0.001) was found in all the knowledge questions between pre and post educational intervention. Likewise, a significant difference (*p* < 0.001) was found in all the attitude questions between pre and post educational intervention.

**Table 3:** Comparison of responses before and after the training on pharmacovigilance regarding knowledge and attitude related questions

Knowledge related questions	Healthcare professionals (n, %)		***p***-value
	Pre-survey correct response (n, %)	Post-survey correct response (n, %)	
Adverse Drug Reaction (ADR) definition	56 (62.9)	89 (100)	< 0.001
Pharmacovigilance (PV) definition	56 (62.9)	84 (94.4)	< 0.001
Most important purpose of Pharmacovigilance	47 (52.8)	86 (96.6)	< 0.001
A serious adverse event in Nepal that should be reported to a regulatory body within	20 (22.5)	79 (88.8)	< 0.001
Rare Adverse Drug Reactions can be identified in the following phase of the clinical trial	32 (36)	78 (87.6)	< 0.001
Which of the following methods is commonly employed by pharmaceutical companies to monitor?	55 (61.8)	79 (88.8)	< 0.001
Do you think ADR reporting is professional responsibility for you	78 (87.6)	89 (100)	< 0.001
The healthcare professionals responsible for reporting ADR in a hospital is/are	82 (92.1)	87 (97.8)	< 0.001
Do you know about the existence of a National PV program in Nepal	39 (43.8)	88 (98.9)	< 0.001
In Nepal, which regulatory body is responsible for monitoring ADR	39 (4.8)	82 (96.1)	< 0.001
Where the international center for ADR monitoring is located	40 (44.9)	86 (96.6)	< 0.001
One of the following agencies in the USA involved in drug safety issues	50 (56.2)	84 (94.4)	< 0.001
**Attitude related statements**	**Pre-Survey Agreement (n, %)**	**Post-Survey Agreement (n, %)**	***p*-value**
Reporting of ADRs as the exploration of mistakes of health professionals	21 (23.6%)	59 (66.3%)	< 0.001
Reporting of adverse drug reaction is necessary	31 (34.8%)	89 (100%)	< 0.001
Establishing ADR monitoring center in every hospital	69 (77.5%)	84 (94.4%)	0.001
Reporting ADR will increase patient safety	39 (43.8%)	89 (100%)	< 0.001

### Assessment of knowledge and attitude mean scores before and after an educational intervention

The comparison of knowledge and attitude mean scores before and after an educational intervention was shown in Table 4. A significant difference (*p < 0.001*) was found in both knowledge and attitude scores.

**Table 4:** Assessment of knowledge and attitude mean scores before and after the educational intervention (*n* = 89)

		Mean ± S.D	Range	***p***-value
**Knowledge**	Total score before educational intervention	6.90 ± 2.527	(1–12)	< 0.001
	Total score after the educational intervention	11.36 ± 1.189	(6–12)	
**Attitude**	Total score before educational intervention	1.80 ± 0.932	(0–4)	< 0.001
	Total score after the educational intervention	3.61 ± 0.556	(2–4)	

### Factors discouraging from reporting of ADRs

**Fig. 1:**
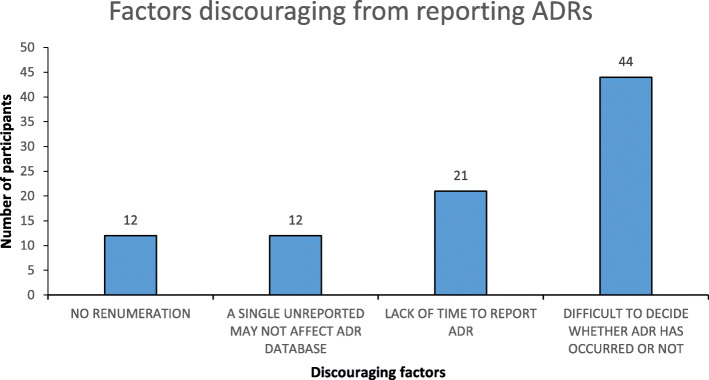
Factors discouraging reporting of ADRs

The factors that discouraged participants from reporting ADR is shown in Fig. 1. Majority of the participants responded to the statement that “Difficult to decide whether ADR has occurred or not”, which was followed by the statement “Lack of time to report ADR”.

### Recommendations for improving ADRs reporting

The participants were asked to provide recommendations for improving ADR reporting and PV system in the future after an educational intervention. The participants provided the recommendations for improving ADRs reporting which was shown in Table 5. Twenty-one (23.6%) of them suggested for frequent PV awareness programs via continuing medical education (CME)/workshop.

**Table 5:** Recommendations for improving adverse drug reactions (ADRs) reporting

Recommendations for improving ADRs reporting	Frequency (n)	Percentage (%)
Frequent pharmacovigilance awareness via continuing medical education (CME) / workshop	21	23.6
Frequent SMS / Email about ADR reporting / Provide toll-free number	3	3.4
Make online reporting of ADRs through software	2	2.2
Keep ADR register in all the wards and Out-patients Department (OPD)	32	36.0
Include ADR forms along with case sheet	11	12.4
Make ADR reporting mandatory	1	1.1
Include ADR column in a medical case sheet	2	2.2
Develop mobile apps for ADR reporting	2	2.2
Discuss ADR cases every month during PV meeting	1	1.1
Make a standardized protocol for reporting ADR	9	10.1
Include ADR reporting in the resident logbook	5	5.6
**Total**	**89**	**100.0**

## Discussion

The present questionnaire-based pre-post intervention cross-sectional study was conducted at a regional PV center at an oncology hospital in Nepal to assess the impact of educational training on HCPs’ knowledge and attitude towards PV. Additionally, this study also assessed the factors that influence ADR reporting among HCPs. Several studies conducted in Nepal that have reported HCPs lack knowledge regarding PV at different hospitals of Nepal [14, 15, 17, 19, 20]. These studies assessed knowledge and attitude without any kind of educational intervention. However, there are only very few limited studies [21, 23], that have assessed the PV knowledge after educational intervention among HCPs. This is the first study to assess the impact of education on PV at a regional PV center of an oncology based hospital in Nepal.

The current study showed females were higher in participation than males. These findings were similar to the studies conducted at different hospitals in Nepal [15, 17, 19, 21], and also similar to studies conducted in different countries such as neighboring countries India [26], and other countries like Japan [32], Iran [33, 34], UAE [35] and Turkey [7]. Most of the participants in this study were nurses and staff nurses. The reason for being more female, staff nurses and nurses is probably due to the preference of females to choose nursing as their career compared to males [14, 17]. Most of the participants were found to be in the age group of 21–30 years with a mean age of 26. The average age of HCPs in present study ranged between 20 and 46 years which is similar to studies [17, 23, 33–35], unlike study conducted at Turkey [7] where the maximum age group was 50–55 years. Almost 90 % of the participants had work experiences of 0.2–5.1 years. In similar other studies [27, 36], participant’s age and work experience have shown an influence of PV activities and ADR reporting. However, in the other studies [36, 37], there was no statistical difference found between ADRs reporting practicing and other independent variables including age, gender, and experience in years.

Majority of participants’ educational qualifications were 48 (53.9%) diploma degree (staff nursing or diploma in pharmacy) which is similar to a study done by Santosh KC et al. [17], conducted at four regional PV centers in Nepal. The findings are also similar to the study by John LJ et al. [35] at UAE, unlike the study conducted by Hanafi et al., wherein, the majority were holding a qualification of bachelor and master degree [34]. The probable reason may be due to the maximum number of diploma-qualified employees in hospitals.

Majority of the participants responded that, difficulty in deciding whether ADR has occurred and lack of time for reporting ADRs. These are the most important factors which discouraged HCPs from reporting ADRs. The similar factors in the present study were also reported by other studies conducted in Nepal [15, 17, 19] and in other countries [35–38]. In studies from other countries, there were several reasons for under-reporting of an ADR was documented, which are: uncertain about ADR, too small and not so serious ADR to report, lack of knowledge about reporting, unavailability of ADR report form. Several studies also reported that: lack of knowledge of the existence of a national PV, regional PV and ADR reporting system, belief that the ADR encountered was previously well recognized, forgetting to report the ADR and ignorance of ADR reporting system [27, 32, 39–42].

In the present study, the majority of the participants suggested increasing ADRs in future such as, “keep ADR register in all the wards and out-patients department”, “frequent pharmacovigilance awareness via continuing medical education (CME) / workshop” and “including ADR reporting forms along with medical case sheet of the patients”. Participants also suggested making an institutionalized protocol for reporting ADRs. These suggestions made by participants were found almost similar to other studies conducted in other countries. This shows that educational interventions and other actions play an important factor in encouraging the increase in ADR reporting and ADR reporting [6, 14, 21, 26, 39, 43–45]. Purposive multidisciplinary collaborative efforts concentrating on a functioning and dynamic upgrade of knowledge concerning PV and ADR, through informative workshops, training, continuing medical educations (CMEs), seminars, online or offline courses, and clinical meets or conferences might rebuild HCPs into better knowledge and awareness of PV along with motivating for ADR reporting practices. This will also increase good practice among HCPs. This is an approach where there is a plentiful extent of improvement of PV and increasing ADRs and should be positively addressed. Drug and Therapeutics Committee (DTC) also plays an active leading role in the advancement of medicine safety and strengthening PV centers at the hospital [46]. Practical training or workshop regarding PV might assist to resolve this up-and-coming problem of underreporting of ADRs at the hospital [44]. A systematic simulated environment and workshop on PV should be conducted for the HCPs (pharmacists, nursing staffs, dentists and even medical doctors) and including regular interaction and interprofessional collaborations to better understand the difficulties they are facing. According to the International Pharmaceutical Federation (FIP) statement of policy, the pharmacist has key responsibilities in monitoring the safety of medicine and a key role in PV and increasing ADR reporting. The pharmacist can work as PV educators to enhance knowledge of HCPs [47]. Similarly, clinical pharmacists or pharmacists also play a vital role in PV such as identifying ADRs, reporting ADRs, counseling regarding ADRs to the patients or HCPs and increasing ADR reporting [48–50].

The knowledge mean score of HCPs was found to be increased after an educational intervention. The findings of this study were found to be similar to several studies conducted in Nepal [14, 17, 19, 21, 23] and other countries [7, 33, 34, 44, 51]. This evidence clearly showed that after the introduction of educational intervention on PV, knowledge of HCPs were improved. Knowledge regarding ADRs and PV is essential to all HCPs and they have an important role in identifying, monitoring, preventing and reporting ADRs. HCPs should have good knowledge of ADR and the process of reporting ADRs to PV center. Among nurses and pharmacists, pharmacists have good knowledge of PV. While, the score was good after the post-study of HCPs working in the hospital which clarifies that PV course, training should be provided to all HCPs in Nepal.

This study revealed that a majority of participants understood that ADR reporting is essential to increase patient safety with drugs. In this study, an attitude score was found to be increased slightly after the educational intervention. This result was found to be similar to other studies [20, 21, 23, 26]. Since the majority of HCPs contemplate ADR reporting is essential, HCPs should overcome hurdles in reporting of ADR and should voluntarily report ADR to regional PV or national PV as their professional responsibility.

Huge improvement in the attitude of participants was noted in this study. This was the main findings of this study, as this regional PV had been newly created and also the first time established at any cancer hospital in Nepal. In the results of pre-training, the attitude was found below. The post-training mean score of attitude was found to be increased than that of the pre-training mean score. These findings are almost similar to other studies [21, 23, 25]. This evidence clearly showed that after the introduction of educational intervention on PV, attitudes of HCPs were improved. This may improve the increase in ADR reporting in the future. In this study, educational intervention is a key component to increase attitude among HCPs was found.

### Strengths and limitations of the study

This study was carried out in one of the oncology-based hospitals in Nepal, with a regional PV center and to assess the pre-testing and post-testing scores of the nurses and pharmacists on PV issues. Still, some limitations might compromise the internal and external validity (i.e., generalizability) of the study findings. The study was a single-centric study with no doctors, medical oncologists as the participants. Also, the changes in the knowledge and attitude score might not be reciprocated into the practice scores and this was not verified. The questionnaire employed might not fully assess the changes in the behavior of HCPs. Moreover, the inherent limitation of cross-sectional study (i.e., conclusive evidence of the causality of the relationship between KAP and associated factors) might be prevalent here as well.

Some initiatives such as follow-ups on ADRs through reminder emails, SMS notification to HCPs can be incorporated into the existing programs. Such programs can be conducted at an interval of about 2–3 months to ensure the persistence of the behavioral changes. Future programs are to be targeted for medical oncologists, doctors and other HCPs to enhance their practice on minimizing ADRs. In the long run, patients must be tied to ADR minimization approaches [52]. Similar kinds of educational interventional studies could be conducted in other hospitals to explore their practice status on ADR minimization.

## Conclusion

In conclusion, the preliminary findings of this current study presented that educational intervention improved the knowledge and attitude among nurses and pharmacists regarding PV and ADR reporting. It also suggests that training on PV and ADRs tends to have a positive impact on knowledge and attitude nurses and pharmacists of the hospital. Anchored in a social posture of learning, the collaboration of all professionals, including the patient implication, will allow the implementation of collaborative best practices to report ADR.

## Data Availability

The datasets used and/or analyzed during the current study are available from the corresponding author on reasonable request.

## Abbreviations

ADR: Adverse drug reactions
DDA: Department of Drug Administration
HCPs: Healthcare professionals
KAP: Knowledge, attitude and practice
NCHRC: Nepal Cancer Hospital Research Center
PV: Pharmacovigilance
